# Harsh Working Conditions and Poor Eating Habits: Health-Related Concerns of Female Head Porters (Kayayei) in the Mallam Atta Market, Accra, Ghana

**DOI:** 10.1155/2018/6201837

**Published:** 2018-02-13

**Authors:** Samuel Harrenson Nyarko, Abdul Majeed Tahiru

**Affiliations:** ^1^Department of Population and Behavioural Sciences, School of Public Health, University of Health and Allied Sciences, Hohoe, Ghana; ^2^Department of Demography, College of Public Policy, University of Texas at San Antonio, San Antonio, TX, USA; ^3^Department of Family and Community Health, School of Public Health, University of Health and Allied Sciences, Hohoe, Ghana

## Abstract

**Background:**

The kaya business is known to pose significant health-related risks to female migrants. This study sought to explore the health-related concerns of female head porters in the Mallam Atta market, Accra, Ghana.

**Methods:**

A qualitative study was conducted in which twenty female head porters at the Mallam Atta market in Accra were interviewed. A thematic analysis was performed and the emerging themes were presented and supported with quotations from the respondents.

**Results:**

Poor accommodation and eating habits, harsh working conditions, and lack of knowledge about health conditions exposed the respondents to several health-related concerns like neck pains, skin rashes, malaria, cholera, and stomach ache among other infections. The popular means of seeking health care was through purchasing drugs from pharmacies or drug peddlers instead of health facilities. Financial constraints, lack of faith in the National Health Insurance Scheme, and long waiting periods at the health facilities militated against seeking appropriate health care at the hospitals and clinics.

**Conclusion:**

Political willpower needs to be strengthened for poverty reduction strategies such as training of hairdressing, dress and soap making, and shea butter processing for women from the Northern regions in order to ameliorate their livelihoods and/or reduce migration to the south.

## 1. Background

Global economic issues such as unemployment, low-income levels, inequitable distribution of resources, forced marriage, family breakdown, lack of education, and peer influence have forced a lot of people to move from their places of origin, mostly rural areas, to major cities instead of staying with their loved ones [1]. Migration has become part of people's lives especially the young people who are mostly in search of greener pastures [2]. In Ghana, the high poverty levels in the Northern parts of the country, namely, the Upper West, Upper East, and Northern Region, compel girls and women to migrate to the southern parts, mostly to the capital cities like Accra and Kumasi, to engage in what is locally called “kaya business” [3].

“Kaya business” refers to the act of carrying loads on the head for a fee and the women who are engaged in this activity are called “Kayayei” (Kayayo for singular). Most of the girls and women who engage in the kaya business or head portage are mainly from the Mamprusi, Gonja, Kotokoli, Mossi, Frafra, Bimoba, and Dagomba ethnic groups which are all located in the Northern parts of Ghana, with a few of the Kayayei from Burkina Faso and Togo [4–6]. Opare [5] and Agarwal et al. [7] assert that most of these female migrants who travel down to the south and to the middle belts are young ladies who are mostly between the ages of 10 and 35 and have limited or no formal education. As a result, these female migrants have no prospects of gaining employment in the formal sector in the cities [8] and, for this reason, they engage in the kaya business as a short-term means of earning income and saving enough money to enter into other profitable ventures in the future [7].

According to Opare [5], some Kayayei engage in the kaya business to acquire possessions like utensils and clothes in the preparations towards marriage while others save money to enable them to undergo an apprenticeship in hairdressing or dressmaking. Some Kayayei want to earn income and send remittances to their families such as husbands, children, and parents back home [3, 9, 10]. When these female migrants arrive at the urban areas, most of them do not have any relations to support them financially. This leads them to settle in slum areas where accommodation comes with a cheap cost and poor quality [11]. Securing such cheap accommodation is one of the survival strategies employed by the Kayayei in the quest to spend little and save more money [7].

In spite of the fact that the kaya business serves as a source of income for most female migrants from Northern Ghana [7, 9], the activity exposes them to various health conditions like diarrhea, malaria, and Sexually Transmitted Infections (STIs). Opare [5], Shamsu-Deen [6], and Kwankye et al. [12] contend that various lifestyle practices such as spending nights in the open and at market centres and eating from unhygienic places which form part of their environment heighten the risk of the Kayayei acquiring most of the aforementioned health problems. The health of the Kayayei is a major concern since, without a healthy mind and body, the Kayayei will not be in a capable position to perform any of their daily tasks of carrying luggage and goods for people, which is their major purpose of being at the market centres [7].

However, some efforts have been made by past governments and some organisations to help resolve some of these health-related concerns. These include the National Health Insurance Scheme (NHIS) which was established in 2004 to provide affordable health care for people. Also, the United Nations Population Fund (UNFPA) and the Society for Women against AIDS in Africa (SWAA), Ghana, have trained and educated some Kayayei on life skills along with sexual and reproductive health education [13]. Nevertheless, most Kayayei still face various health-related challenges. For instance, Van den Berg [1] and Alfers [10] explain that some Kayayei are unable to afford the NHIS premium, which means they cannot benefit from the scheme. Alfers [10] further argues that, apart from their inability to pay for the NHIS premium, most Kayayei complain that the scheme does not work when used in accessing health care. A considerable number of studies have examined issues on female head portage, most of which focused on the livelihood and survival strategies of female head porters [1, 3, 14]. However, the public health needs and the health implications of the activities and surviving strategies of the female head porters have been less explored [6, 15]. For instance, little is, therefore, known about the health-related concerns and health-seeking behaviour of the female head porters. Consequently, we explore the health-related issues of the Kayayei in the Mallam Atta market in Accra including their health-seeking behaviour and the challenges faced when seeking health care. We, therefore, seek to ask and provide answers to the following research questions: what are the factors that expose the Kayayei to health-related problems? What is the pattern of health-seeking behaviour among the Kayayei and what are the challenges the Kayayei face when seeking health care?

## 2. Materials and Methods

### 2.1. Study Population and Design

The target population of this study was the Kayayei at the Mallam Atta market in the Accra Metropolis, Ghana, and its surroundings. This study adopted a qualitative research design. This is to give the respondents the opportunity to construct their own meanings to the situation and to make sense of their personal experiences [16]. This design was used in order to generate an in-depth knowledge on the health-related concerns of the Kayayei in the Mallam Atta market.

### 2.2. Sample and Sampling Procedure

Purposive and convenience sampling techniques were used to select the respondents for the study. With this, female migrants who were believed to be Kayayei were recruited for the study. Since the majority of them work during the greater part of the day and are almost always on the lookout for potential customers, only those who were readily available at the time of the study were recruited to the study. The number of respondents who participated in the study was determined based on thematic saturation. Thus, respondents were continuously recruited until no new data were generated. Following this, we achieved total saturation of themes after recruiting twenty (20) respondents.

### 2.3. Data Collection

Data for the study were collected through in-depth interviews with individual respondents. These were done at convenient periods when the Kayayei were having their respite during working days and when they finished work. The interviews were conducted on a face-to-face basis with the use of an in-depth interview guide which contained semistructured questions categorised into four sections such as demographic information, factors that expose Kayayei to health-related issues, health-seeking behaviour, and the challenges they faced when seeking health care. Questions were probed further in order to explore the individual experiences and different opinions among respondents. Most of the interviews were conducted using the Twi language as most of the respondents could not read or speak the English language. However, a few of the participants opted to be interviewed in Pidgin English. In the course of the data collection process, the interviews were individually tape-recorded and the audios were saved for further processing and analysis. Prior to the study, an ethical clearance was obtained from the Ghana Health Service Ethical Review Committee (GHS/ERC). A written informed consent was sought and respondents were assured of confidentiality and anonymity of information while only respondents who were willing to participate were included in the study.

### 2.4. Data Processing and Analysis

The audios generated from the tape-recorded interviews were played a number of times to get the general understanding of the information contained in them. The tape recordings were then carefully translated from Twi and Pidgin English into the proper English language and subsequently transcribed into individual scripts based on the emerging themes from the interviews. To ensure data quality, the transcripts and the audios were double-checked by a language expert in Twi and English to make sure that the transcripts were a true representation of the information presented in the interviews.

The data processing and analysis were performed manually. In doing this, the transcripts were read a number of times to get familiarised with the content and then the transcripts were proofread and edited for errors and omissions. While reading the transcripts, the emerging themes were identified and sorted for all the interviews. Under each emerging theme, the opinions of all the respondents were identified and captured and then presented in line with the research questions of the study. In presenting the results, the views of respondents were presented in the form of quotations to support the various emerging themes. These were carefully analysed and discussed in relation to the available literature on the subject.

## 3. Results

### 3.1. Demographic Characteristics of Respondents

The respondents were between 15 and 30 years old. They came from areas like Walewale, Bilen, Wa, Janga, Saboba, Sandema, and Yaloma among others in the Northern sector of Ghana. Most of them had little formal education ranging from primary school to junior high school education or no formal education. Apart from the kaya business, some of them also engaged in other activities like washing and cleaning clothes in households for a fee, washing plates for food vendors, and sewing among others. The respondents also reported that the amount of money they earned daily ranged between GhC 10 ($2.30) and GhC 25 ($5.60).

### 3.2. Factors Exposing Respondents to Health-Related Concerns

The kaya business offers the respondents the opportunity to earn their living. However, this study has found that a number of factors such as poor accommodation and sanitation facilities, poor eating habits, harsh working conditions, and ignorance exposed the respondents to various health problems like malaria, cholera, skin rashes, and frequent headaches among others. Poor accommodation and sanitation facilities were some of the challenges to the health of the respondents. They reported sleeping in a wooden or “tent-like” (layers of cement blocks arranged on top of each other by the respondents themselves without doors) structures which they shared as their accommodation with their colleagues. They reported that they kept their belongings like clothes, pans, and money among others in their makeshift structures. Some of them also reported that they slept outside these makeshift structures but kept their belongings inside because the room was small and congested. For instance, one of them said the following:

*I sleep outside with some of my colleagues because the room is full so we all cannot sleep in it. However, my clothing and everything is in the room.* (Respondent 2, 17 years)

Another respondent also added the following:

*When the rain falls, we go to sleep or stand on the veranda of the provision stores or drinking spots. And mosquitoes bite us there a lot.* (Respondent 6, 17 years)

This was reported by fifteen respondents who explained that they slept outside the makeshift structures but kept their belongings inside. These respondents reported that those who slept inside these crowded structures ever reported contracting illnesses such as skin rashes and coughs which spread quickly among them. One respondent said the following:

*The place I sleep is somewhat okay, but there are some small black insects that bite us there and it itches a lot. Even as I speak now, there are rashes all over the body of me and my child because of the insect bites.* (Respondent 15, 19 years)

One of the respondents who slept in the makeshift structures also reported the following in relation to their accommodation and its possible health implications:* “There are a lot of mosquitoes in these tents and structures which give us malaria”* (Respondent 8, 21 years). The poor accommodation patterns also posed sanitation challenges to the respondents. The makeshift structures used by the respondents had no toilet and/or bath. As a result, the respondents used public toilets and bathrooms. For example, one of them described the state of the public toilet and bathroom facilities as sickening and smelly in the following statement:

*I pay money to use public bathroom and toilets. The public bathrooms and toilets are not hygienic and they smell a lot, but what can I do?* (Respondent 7, 18 years)

Another issue that came out of the interviews was poor eating habits. All respondents reported that they did not cook and bought foods from the roadside or around the market centres which were potentially unhygienic. It was believed that the eating habits of the respondents had a negative impact on their health. One of them described her eating pattern as follows:

*I know it is better to cook at home than to buy from the roadside since I am not aware of how the food I buy is prepared and the place where I buy it is not hygienic either. However, I buy food from the roadside because it is less expensive.* (Respondent 10, 22 years)

Some respondents also added the following:

*I sometimes cook when I have money but mostly I buy from the roadside because cooking is very expensive as compared to buying from the roadside.* (Respondent 4, 23 years)

*I always buy food from outside because there is no place to cook. Besides, I do not have cooking utensils here to enable me to cook for myself.* (Respondent 9, 21 years)

Another issue which emerged was the harsh working conditions experienced by the respondents. The respondents reported that the kaya business was very tedious and affected their health negatively. One respondent reported the following when she was asked whether the kaya business had ever affected her health in any way:

*Yes, sometimes, I carry heavy loads and walk long distances under the scorching sun and then my chest and neck will be paining me, but it does not fetch enough money too.* (Respondent 3, 30 years)

Another respondent also replied with the following:

*I get chest pains and illness as a result of the heavy loads I carry, and because I walk under the sun for a very long time with my baby strapped to my back, it makes the situation worse*. (Respondent 8, 21 years)

Furthermore, it came out that ignorance on the part of some respondents about possible health risks posed by their living conditions was a problem. The respondents reported that even though they knew that overcrowding in the room exposed them to a number of health problems, they did not know the names of the specific illnesses they could contract. For instance, when one respondent was asked whether she thinks the overcrowding in the room can spread certain illnesses, she responded with the following:

*Yes, but sometimes we do not know which of the illnesses could be transmitted to us*. (Respondent 20, 22 years)

However, some of these respondents revealed that, back in their hometowns, they hardly got lice in their hair, but when they came to Accra they got them. They explained that it is very common to see their colleagues picking lice from each other's hair, and this may be the result of overcrowding and poor personal hygiene among them.

### 3.3. Health-Seeking Behaviours of the Respondents

One of the objectives of this study is to explore the health-seeking practices of the respondents. With regard to this, a number of themes emerged which formed the health-seeking behaviours adopted by respondents when they had health problems. It was found that the majority of the respondents visited drug stores and other drug peddlers when they were ill. Out of the 20 respondents, 15 reported that whenever they fell sick, they bought drugs from the drug stores and drug peddlers (individuals who hawk drugs at the market centre). For instance, one respondent reported the following:

*When I fall sick, I go to the drugstore and describe the symptoms of the illness to the chemist and the person prescribes a drug for me.* (Respondent 5, 22 years)

Moreover, out of the 15 respondents who patronised drug stores and drug peddlers, 10 preferred buying drugs from the peddlers to the pharmacies. One respondent, therefore, explained why she preferred drug peddlers to pharmacies or drug stores:

*I buy my drugs from the drug peddlers because it is cheaper and more expensive when I buy it from the pharmacy. Besides, there is no money these days, and I want to save more money in order to go back to my hometown.* (Respondent 14, 21 years)

Another respondent also explained why she preferred drugstores to hospitals:

*When I buy from the drug stores, I get better, so I do not see why I should go to the hospital or clinic to go and join long queues and pay a huge sum of money.* (Respondent 5, 19 years)

It was further found that only a few respondents (5) preferred visiting health facilities (hospitals and clinics) when they were sick. They expressed their preference for receiving health care at the hospital but further explained that most often some of them could not afford the hospital due to their low income. One of them, for instance, said the following:

*I know very well that the best place to go when I am sick is the hospital, but sometimes, money is the problem.* (Respondent 14, 25 years)

Even though they reported that going to health facilities for medical attention was the best, only three reported that they actually went to the hospital when they were sick. One of them explained the following:

*I go to the hospital when I fall sick. I use part of the money I earned from the kaya business to pay for my health care.* (Respondent 4, 28 years)

They further explained that they attended the health facilities every time they felt sick and explained why they preferred going to the hospital as compared to buying drugs from the drug peddlers and the pharmacies:

*Sometimes, when you purchase drugs from the drug stores and the drug peddlers, the drugs do not seem effective. But whenever I visit the hospital, I am well taken care of and the doctor might even inject me so I get better faster, that is why I prefer hospitals to the pharmacy shops and drug peddlers.* (Respondent 4, 23 years)

However, two respondents indicated that they neither bought drugs nor attended hospitals or clinics when they fell sick. They explained that whenever they fell sick, they just tried to take a rest and then automatically recuperate without taking any specific action. One of them reported the following:

*When I am sick, I do not do anything because I do not have enough money to go to the drugstore to buy medicine or go to the hospital. I just lie down and sleep, and hope to gradually recuperate. So if I am sick, I neither go to the hospital nor the drug store*. (Respondent 19, 20 years)

This is a major indication that the majority of the respondents resorted to drug stores and drug peddlers while only a few attended hospital when they fell sick.

### 3.4. Challenges to Health-Seeking Behaviours of Respondents

The study also sought to explore the challenges that affect the health-seeking behaviour of the respondents. It, therefore, emerged that inadequate income and long hours of waiting at the health facilities were the major factors that negatively affected the appropriate health-seeking behaviour of the respondents. Seventeen out of the 20 respondents reported that inadequate earnings were a major reason why they could not seek appropriate health care from the health facilities. When asked why they did not seek appropriate health care when they fell sick, the following was the response of two of the respondents:

*I wish I could go to the hospital anytime I am not well, but I do not have enough money to do so.* (Respondent 7, 18 years)

*If money to pay for the cost of treatment at the hospital is not available, I have to sit at home with the illness. So, I buy drugs from drug peddlers because hospitals do not reduce the prices of their drugs, unlike the drug peddlers. Besides, I do not have enough money.* (Respondent 13, 22 years)

The respondents were asked whether they used the National Health Insurance Scheme (NHIS) since they did not earn enough income to pay for the cost of treatment at the health facilities. Even though a number of them reported that they used the scheme, most of them further explained that having the NHIS did not prevent them from buying drugs. This is because some of the drugs prescribed at the hospitals can only be found outside the health facilities in pharmacies that are not NHIS-accredited, which means that patients will have to personally pay for their drugs. This is evident from the following reports of two respondents:

*If you go to the hospital with NHIS, they will tell you to go and buy some of the drugs outside and if you do not have the money, you cannot buy the drugs. *(Respondent 7, 18 years)

*I did my NHIS in my hometown, and they told me I can use it everywhere, but here in Accra, whenever I take it to the hospital, they tell me to pay before they treat me. *(Respondent 20, 22 years)

Another emerging challenge with respect to seeking appropriate health care at the hospitals was the long waiting hours at the health facilities. The respondents who utilised the health facilities reported waiting for longer periods of time before attending. This, they explained, was due to congestion at the health facilities and sometimes the late arrival of some medical doctors. Some of the respondents who attended hospital when they were sick said the following:

*There are always a lot of people at the hospital and I have to wait for a very long time before they attend to me*. (Respondent 4, 23 years)

*At times, the doctors come in late, so after taking your card and going to see the nurses, you have to wait in a long queue until the doctor comes.* (Respondent 7, 15 years)

According to the respondents, these were the main factors that militated against their seeking of appropriate health care whenever they and their children fell sick.

## 4. Discussion

The findings of this study show that majority of the Kayayei live in makeshift structures made of wood, cement blocks, and tents as their accommodation. These structures do not have facilities such as toilets, bathrooms, and potable water. This is generally similar to the accommodation arrangement of the Kayayei as described by Van den Berg [1], Agarwal et al. [7], and Kwankye et al. [12]. The accommodation structures of the Kayayei are clustered around the Mallam Atta market and surrounded by a lot of filth. For those who sleep outside, they are exposed to malaria since the choked gutters around the marketplaces breed a lot of mosquitoes. The findings of this study confirm the findings of Agarwal et al. [7] and Taylor et al. [17] that the accommodation arrangements of Kayayei heighten their risk of diseases like skin rashes, malaria, and cholera among others.

It was also observed that the earnings per day of the Kayayei were low as they ranged from GhC 10 ($2.30) to GhC 25 ($5.60). With such a low daily income, the Kayayei could not afford to rent a decent accommodation. As a result, most Kayayei reside in slum areas around the Mallam Atta market where they complain that the public toilets and bathrooms are unhygienic and appalling. This is consistent with the observation of Tufuor [11] and Taylor et al. [17] who assert that the urban poor including most Kayayei usually reside in slum areas without sanitation facilities since they cannot afford to pay for high cost of rent in urban areas. This situation is further worsened by inadequate potable water to maintain personal hygiene and consequently increase the risk of contracting infectious diseases. However, the study shows that these accommodation arrangements of the respondents did not expose them to sexual harassments and rape from men as observed by Boateng and Korang-Okrah [3] and Shamsu-Deen [6].

Another factor that exposed the Kayayei to health-related problems was poor eating habits. Buying food from the roadside and around the Mallam Atta market was the eating pattern reported by all respondents. The Kayayei did not cook because of inadequate income and because it seems comparatively cheaper and convenient to buy food than to cook. Even though these were the main reasons reported by the respondents, one can easily deduce that one of the main reasons for buying food from the roadside is because they lack cooking spaces and, besides, they work through the greater part of the day and hence have inadequate time to prepare meals for themselves. This is because most respondents reported that they start work at 5 am and finish at 7 pm. In effect, it is not the issue of purchasing food from the roadside that is bad in itself; instead, the problem emanates from the unwholesome environment in which these foods are prepared, purchased, and eaten. Boateng and Korang-Okrah [3] and Yeboah [18] also made a similar observation where the Kayayei usually buy foods and eat from market centres which are prepared under unhygienic conditions.

Harsh working conditions of the kaya business also exposed the Kayayei to health problems like headaches, chest pains, neck pain, and general body pains among others. It is evident from this study that the loads carried by the Kayayei were heavy. Van den Berg [1] and Yeboah [18] argue that the kaya business is very tedious because the Kayayei use more physical energy when transporting goods from one place to the other at the market centres. The loads carried are heavy while the market centres where the Kayayei operate are usually crowded. Consequently, the Kayayei have to find a way to meander their way through these crowded market centres while carrying heavy loads for their customers; hence they experience constant general body pains. Additionally, ignorance about the specific health-related implications of the conditions in which they live was a potential major problem for the Kayayei. Ignorance about these health-related issues may be the result of lack of formal education among the Kayayei. Aside from the lack of formal education, the nature of the business does not always offer them the opportunity to get adequate information from the right sources on their health concerns. For instance, some Kayayei believe that they are so close to each other; therefore, they share some personal items including pins and needles for their tattoos, but they were unaware that sharing these items can transmit infections such as HIV/AIDS from one person to the other. This situation implies a high level of ignorance among most of the Kayayei which may be quite deleterious to their health.

With regard to their health-seeking behaviours, it came out that the major way by which the Kayayei seek health care is by visiting the pharmacy and/or drug peddlers. At the pharmacy or the drug peddler's place, the Kayayei describe their health problem to the pharmacist or the drug peddler who in turn recommends drugs for them. The main reason given for using this method of seeking health care instead of attending a health facility is that it is cheaper to buy from the drug stores and drug peddlers compared to attending the hospital. The drug peddlers are mostly their first point of call when they fall sick and if the illness persists then they go to the pharmacy. It is observed that the main pattern of seeking health care among the Kayayei usually starts from drug peddlers to the pharmacies and then to the health facilities as a last resort. Shamsu-Deen [6] and Kwankye et al. [12] also observed that the Kayayei go to pharmacies when they are sick and do not utilise health facilities when they are confronted with health problems. However, the phenomenon of seeking health care from drug peddlers and drugstores may be dangerous to the health of the Kayayei, as most of these drug peddlers and drugstore attendants are not trained pharmacists and may end up giving wrong prescriptions which can compound the health conditions of the Kayayei.

Another health-seeking method used by some Kayayei is the attendance of hospitals for treatment. In this study, it is found that only a few of the Kayayei attend hospitals or clinics when they fall sick because of financial challenges which constrain their payment for treatment. A few of them use the little earnings from the head portage business to access health services at the health facilities whereas a few also become apathetic and take no health-seeking action when they fall sick. With this strategy, they just attempt to take a rest for a while until they get better or feel well enough to resume work.

Furthermore, it emerged from the study that the Kayayei face some challenges when seeking health care, especially from the health facilities. The major challenge faced by Kayayei when seeking health care is financial constraints. The average money earned by the Kayayei range from GHC 10 ($2.30) to GHC 25 ($5.60). It is usually from these same daily earnings that they buy food, pay for the use of public sanitation facilities (toilet and bathroom), save money, and send remittances back home. As such, seeking appropriate health care was negatively affected by the low earnings of the Kayayei. Most Kayayei could not have access to health services at hospitals and clinics because they could not afford it. Even though some Kayayei prefer to receive health care at the health facilities, only a few are able to do so due to financial challenges. This confirms Asaana's [19] argument that seeking health care is expensive for female migrants. The preference for health facilities among the Kayayei shows that if they earn more, they may seek better health care [19]. One can observe that the problem of finance is a challenge which has been at the centre of almost all the challenges faced by the Kayayei. Thus, the factors that expose them to health problems and their health-seeking behavior as well as the challenges they face when seeking health care, all, hinge on the issue of inadequate finance.

Another challenge that the Kayayei face when seeking health care has to do with the coverage and the effectiveness of the NHIS. The Kayayei explain that the NHIS does not cover the essential drugs that are expensive. Thus, not all essential drugs are covered by the scheme; hence, the Kayayei still need money to pay for proper health care even when they have a health insurance card. This echoes what Dalinjong and Laar [20] observe: certain procedures in accessing health care have not changed even with the introduction of the NHIS, which include essential drugs not covered by the NHIS. The NHIS cards of some Kayayei which they acquired from their hometowns could not be accepted at health facilities in Accra. For these reasons, women in the informal sector usually do not trust the efficacy of the NHIS [10], and this has developed negative perceptions about the use of the scheme for seeking appropriate health care. Finally, long waiting periods at the health facilities had also negatively affected the attendance by the Kayayei. They believe that the long waiting hours which are caused by long queues and the late arrival of some medical personnel may waste their productive day and they may end up earning nothing for the day. Hence, they will rather patronise drug stores and drug peddlers instead of seeking appropriate health care at the health facilities.

## 5. Conclusions

Even though the head portage business provides the Kayayei with their livelihood, poor accommodation and eating habits, harsh working conditions, and ignorance about health conditions expose them to myriads of health problems like neck pains, skin rashes, malaria, cholera, and stomach ache among other infections. The majority of Kayayei try to seek health care from the drugstores or drug peddlers around the market centres, instead of appropriate health care from the health facilities, due to affordability and convenience. Financial constraints, lack of faith in the NHIS, and long waiting periods at the health facilities are the key factors that militate against appropriate health-seeking behaviour among the Kayayei. Following from this, new research issues can focus on examining the physical, social, and mental health status of the Kayayei in order to reveal the state of their health needs and to provide relevant interventions to deal with them. Also, strong political willpower that will seek to develop and implement relevant policies to ameliorate the livelihood conditions of the Kayayei is crucial.
